# Vitamin D Decreases Plasma Trimethylamine-N-oxide Level in Mice by Regulating Gut Microbiota

**DOI:** 10.1155/2020/9896743

**Published:** 2020-10-05

**Authors:** Xin Wang, Xueqi Li, Yumei Dong

**Affiliations:** The Fourth Affiliated Hospital of Harbin Medical University, Harbin, China

## Abstract

As a metabolite generated by gut microbiota, trimethylamine-N-oxide (TMAO) has been proven to promote atherosclerosis and is a novel potential risk factor for cardiovascular disease (CVD). The objective of this study was to examine whether regulating gut microbiota by vitamin D supplementation could reduce the plasma TMAO level in mice. For 16 weeks, C57BL/6J mice were fed a chow (C) or high-choline diet (HC) without or with supplementation of vitamin D_3_ (CD3 and HCD3) or a high-choline diet with vitamin D_3_ supplementation and antibiotics (HCD3A). The results indicate that the HC group exhibited higher plasma trimethylamine (TMA) and TMAO levels, lower richness of gut microbiota, and significantly increased Firmicutes and decreased Bacteroidetes as compared with group C. Vitamin D supplementation significantly reduced plasma TMA and TMAO levels in mice fed a high-choline diet. Furthermore, gut microbiota composition was regulated, and the Firmicutes/Bacteroidetes ratio was reduced by vitamin D. Spearman correlation analysis indicated that *Bacteroides* and *Akkermansia* were negatively correlated with plasma TMAO in the HC and HCD3 groups. Our study provides a novel avenue for the prevention and treatment of CVD with vitamin D.

## 1. Introduction

According to the World Health Organization, cardiovascular disease (CVD) is the principal risk factor for mortality in many developed and developing countries and is expected to cause approximately 23.6 million deaths worldwide by 2030 [1]. In recent years, gut microbiota has attracted substantial attention due to its association with CVD, and gut microbiota metabolites have been proven to participate in the host's physiological processes and the pathogenesis of metabolic diseases [2]. Trimethylamine-N-oxide (TMAO) is a metabolite generated by gut microbiota. Choline, L-carnitine, and phosphatidylcholine in red meat, eggs, fish, and high-fat dairy products are catabolized by gut microbiota into trimethylamine (TMA), which is upon entering the liver via the portal circulation and being further oxidized by flavin-containing monooxygenases (FMOs), particularly FMO3, to TMAO [3]. Choline is an essential nutrient that plays an important role in lipid transport, cell membrane signaling, neurotransmitter synthesis, and methyl group metabolism. Although choline can be synthesized endogenously, it is not sufficient to meet human needs. Therefore, adequate dietary intake of choline is a necessary condition to maintain human health. The function of choline as a nutrient is a potential double-edged sword. It has been reported that supplementation with a high-choline diet in mice promoted the upregulation of multiple macrophage scavenger receptors associated with atherosclerosis (AS) [4]. In addition, the involvement of choline and its metabolite TMAO in endothelial dysfunction and AS has been repeatedly demonstrated.

Both clinical and animal studies have demonstrated that TMAO can promote AS and is an important independent predictor of CVD [5]. In a large clinical study involving 4,007 individuals, it was observed that a raised plasma TMAO level was related to a higher risk of major adverse cardiovascular events including myocardial infarction, stroke, or death, and following adjustment for traditional risk factors, the relationship still remained significant [6]. TMAO has been shown to inhibit reverse cholesterol transport by decreasing the expression of key enzymes CYP27A1 and CYP7A1 and induce foam cell formation by increasing the expression levels of scavenger receptors CD36 and SR-A1 on the surface of macrophages to promote the development of AS in apolipoprotein E-knockout (*ApoE^−/−^*) mice [7]. Moreover, TMAO can enhance platelet aggregation and promote thrombosis by increasing calcium release from the endoplasmic reticulum of platelet cells [8]. Remarkably, deleting gut microbiota using broad-spectrum antibiotics eliminates the production of TMAO and decreases AS in *ApoE^−/−^* mice [9]. Therefore, CVD could be effectively prevented or treated by reducing plasma TMAO, and gut microbiota is critical to the pathogenesis of AS induced by TMAO and may become an important regulatory target.

Vitamin D is an important fat-soluble vitamin that is primarily known for its regulatory function of calcium and phosphorus metabolism and maintenance of bone health. In recent years, the role of vitamin D in cardiovascular health has attracted increasing attention. Approximately 70% of the world's population suffers from vitamin D deficiency or insufficiency due to inadequate sunlight exposure (especially ultraviolet B) and changes in dietary composition [10]. Clinical research has shown that a lack of vitamin D leads to an increased risk of AS and resulting CVD, such as myocardial infarction, coronary heart disease, or heart failure, which may be linked to inflammatory response, vascular calcification, impaired endothelial function, myocardial cell hypertrophy, and dysregulation of the renin-angiotensin system [11–13]. Although the mechanism is not yet fully understood, deficiency in vitamin D is regarded as a potential risk factor for CVD. Animal studies have demonstrated that oral supplementation with vitamin D can suppress chronic inflammation in the arterial wall and reduce AS by changing the differentiation or function of regulatory T cells and dendritic cells [14]. There also exists evidence in mice that vitamin D may influence the gut microbiota composition and function via the induction of antimicrobial peptide gene expression [15]. Therefore, we hypothesize that vitamin D may be an important regulator of the effect of gut microbiota metabolites on AS and the resulting CVD. The aim of the present study was to explore whether vitamin D supplementation can regulate gut microbiota and thus reduce plasma TMAO level.

## 2. Materials and Methods

### 2.1. Animals and Dietary Treatment

Fifty C57BL/6J mice (female, 6−8 weeks, procured from Beijing Vital River Laboratory Animal Technology Co., Ltd.) were housed under a strict 12 h/12 h light/dark cycle at ambient temperature (18−24°C) and comfortable humidity (50–60%). The initial body weight was 18.2 ± 1.1 g. The mice were provided with sufficient water and food. The food intake and body weight of the mice were recorded weekly over the experimental period. The Ethics Committee of Harbin Medical University, Harbin, China, approved all the experimental procedures.

Following acclimation to the new environment for seven days, mice were allocated randomly to five groups of 10 mice: (1) control group (C), fed a chow diet (AIN93G diet containing 1000 IU vitamin D_3_/kg diet); (2) high-choline group (HC), fed a chow diet plus 1.0% choline; (3) vitamin D_3_ group (CD3), fed a chow diet supplemented with 2000 IU vitamin D_3_/kg diet; (4) high choline+vitamin D_3_ group (HCD3), fed a high-choline diet supplemented with 2000 IU vitamin D_3_/kg diet; and (5) antibiotic group (HCD3A), fed a high-choline diet supplemented with 2000 IU vitamin D_3_/kg diet and given drinking water containing broad-spectrum antibiotics (1 g/L ampicillin, 1 g/L metronidazole, 1 g/L neomycin sulfate, and 0.5 g/L vancomycin). After 16 weeks of treatment, mice were euthanized following a 12 h fasting period. Serum was collected by centrifugation and maintained at −80°C. The liver and the cecal contents were immediately collected and stored in liquid nitrogen at −80°C until future use.

### 2.2. Biochemical Parameter Measurements

Commercial reagent kits (purchased from Biosino, Beijing, China) were utilized to measure the serum total cholesterol (TC), triglyceride (TG), low-density lipoprotein cholesterol (LDL-C), and high-density lipoprotein cholesterol (HDL-C) levels. An ELISA kit (Abcam, Cambridge, UK) was employed to measure the level of serum 25-hydroxy vitamin D (25(OH)D). The levels of plasma TMA and TMAO were measured by stable-isotope-dilution liquid chromatography coupled to triple-quadrupole mass spectrometry (Agilent 6460, California, USA), using d9-TMA and d9-TMAO as internal standards as described previously [16]. Different concentrations of the standards were used to generate calibration curves with which to quantitate plasma TMAO and TMA levels.

### 2.3. RT-qPCR and Western Blotting

Total RNA extraction from frozen liver tissue was performed using the RNAiso Plus reagent (Takara, Dalian, China). Total RNA (100 ng) was reverse-transcribed to cDNA with the aid of the PrimeScript™ RT reagent kit (Takara, Dalian, China). Duplicate RT-qPCR reactions were performed using FMO3 primers (5′-GGAACCAGGAATATGGAAG-3′ and 5′-GGTGACCTTCTGAGCTACAT-3′) and the TB Green® Premix Ex Taq™ II (Takara, Dalian, China) on the Applied Biosystems 7500 Real-Time PCR System (Applied Biosystems, Foster City, CA, USA). The reaction conditions were an initial denaturation at 95°C (30 s), followed by 40 cycles of denaturation at 95°C (5 s) and annealing at 60°C (35 s). The relative mRNA expression level of FMO3 was determined by the 2^-*ΔΔ*Ct^ method using GAPDH as the internal control.

Liver tissue was homogenized in lysis buffer containing protease inhibitors, following which the bicinchoninic acid (BCA) method was employed to determine protein concentration. SDS-PAGE (12%) was utilized to separate proteins, which were subsequently blotted to the PVDF membrane. Following blockade of nonspecific sites with 5% milk in PBS, the membranes were incubated overnight with a primary antibody (rabbit anti-FMO3, Abcam, Cambridge, UK). The next day, after being washed in TBST, the membranes were incubated with the corresponding secondary antibody (anti-rabbit IgG, Abcam, Cambridge, UK). Protein bands were visualized by enhanced chemiluminescence and quantitated using the Image Lab™ software (Bio-Rad, CA, USA).

### 2.4. Illumina MiSeq Sequencing

As instructed by the manufacturer, the PowerSoil® DNA Isolation kit (MoBio Laboratories, CA, USA) was employed to extract microbial DNA from cecal contents. The V4 region of the bacterial 16S rRNA gene was amplified using barcoded primers 515F (5′-GTGCCAGCMGCCGCGGTAA-3′) and 806R (5′-GGACTACHVGGGTWTCTAAT-3′). The PCR conditions were an initial denaturation for 3 min at 95°C, followed by 25 cycles of denaturation at 95°C for 30 s, annealing at 55°C for 30 s, and extension at 72°C for 30 s, in addition to a final elongation at 72°C for 7 min. The AxyPrep DNA Gel Extraction kit (Axygen, Hangzhou, China) was applied to purify PCR products, and standard protocols were followed to sequence purified amplicons on the Illumina MiSeq platform. Raw sequence data was quality-filtered using the QIIME software package (version 1.7.0), and USEARCH was used to cluster high-quality representative sequences into OTUs (operational taxonomic units) based on 97% identity against the Greengenes reference database.

### 2.5. Statistical Analysis

The SPSS 18.0 software was employed for all statistical analyses. The data were represented as the mean ± standard deviation (SD) and analyzed by one-way ANOVA. Group differences were investigated by Tukey's multiple comparison test. Principal coordinates analysis (PCoA) was conducted based on unweighted UniFrac distance matrices to evaluate differences in the gut microbiota community structure among dietary treatments. The linear discriminant analysis (LDA) effect size (LEfSe) analysis was conducted to detect differentially abundant bacterial taxa between groups. *P* < 0.05 was considered statistically significant.

## 3. Results

### 3.1. Food Intake, Body Weight, and Serum Parameters

The effect of the treatments on food intake, body weight, and serum TC, TG, LDL-C, HDL-C, and 25(OH)D levels is depicted in Table 1. No statistical differences were detected in terms of food intake, body weight, or serum lipid parameters (including TC, TG, LDL-C, and HDL-C) among the five groups (*P* > 0.05). However, the serum 25(OH)D level was much higher in the groups supplemented with vitamin D_3_ (CD3, 62.03 ± 9.26 ng/mL; HCD3, 59.45 ± 4.38 ng/mL; and HCD3A, 51.07 ± 8.02 ng/mL) than both the control (C, 26.38 ± 3.41 ng/mL) and high-choline (HC, 27.71 ± 5.95 ng/mL) groups (*P* < 0.01).

### 3.2. Plasma TMA and TMAO Levels

**Figure 1:**
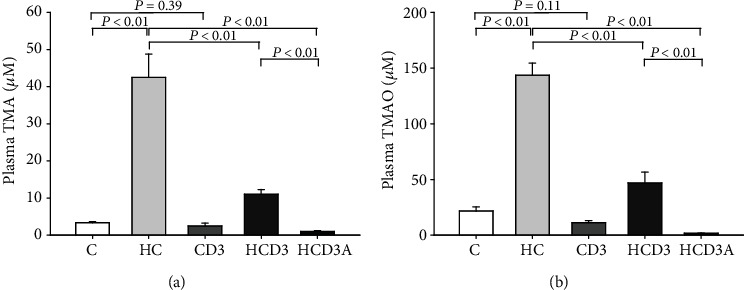
Effects of a high-choline diet and vitamin D_3_ supplementation on plasma TMA and TMAO levels: (a) TMA level; (b) TMAO level. Chow diet (C, *n* = 9), high-choline diet (HC, *n* = 9), chow diet supplemented with vitamin D_3_ (CD3, *n* = 10), high-choline diet supplemented with vitamin D_3_ (HCD3, *n* = 8), and high-choline diet supplemented with vitamin D_3_ and broad-spectrum antibiotics (HCD3A, *n* = 9) were fed to C57BL/6J mice for 16 weeks. Data are represented as the mean ± SD. Significant differences between groups were determined by one-way ANOVA.

After 16 weeks of treatment, dietary choline significantly increased the level of plasma TMA in group HC as compared with the C group (*P* < 0.01; Figure 1(a)). The plasma level of TMA in the HCD3 group was 11.09 ± 1.21 *μ*M, which was much lower than that in the HC group (42.52 ± 6.27 *μ*M, *P* < 0.01). No significant difference was found between groups C and CD3 (3.36 ± 0.29 *μ*M vs. 2.54 ± 0.68 *μ*M, *P* = 0.39). While the TMAO level was substantially elevated in mice fed a high-choline diet, vitamin D_3_ supplementation markedly inhibited this increase (C, 21.81 ± 3.58 *μ*M; HC, 143.74 ± 10.86 *μ*M; and HCD3, 47.03 ± 9.52 *μ*M, *P* < 0.01; Figure 1(b)). Additionally, the antibiotic-treated group (HCD3A) had significantly lower plasma TMA and TMAO levels than the HC group (1.01 ± 0.23 *μ*M vs. 42.52 ± 6.27 *μ*M, 1.76 ± 0.19 *μ*M vs. 143.74 ± 10.86 *μ*M, *P* < 0.01).

### 3.3. FMO3 Protein and mRNA Expression

**Figure 2:**
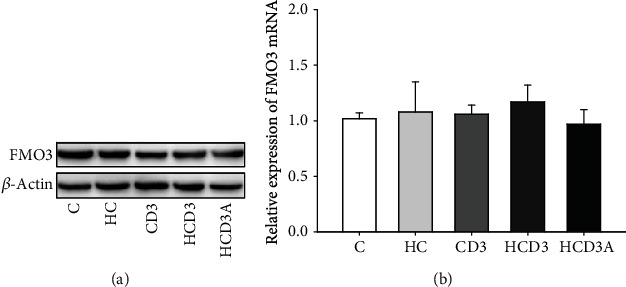
Analysis of FMO3 protein and mRNA expression in the liver. (a) Western blotting of FMO3 protein expression normalized to the *β*-actin loading control; (b) RT-qPCR quantitation of FMO3 mRNA expression. Data are represented as the mean ± SD (*n* = 5).

Plasma TMAO is mainly converted from TMA by liver FMO3. Western blotting and RT-qPCR were utilized to assess whether vitamin D_3_ supplementation regulates the expression of FMO3 at the protein and mRNA levels, respectively. There was no significant difference in the protein expression level of liver FMO3 among the groups (*P* > 0.05; Figure 2(a)). Although the expression of FMO3 mRNA in the HCD3 group was slightly higher than that in the HC group, the difference was not statistically significant (*P* = 0.27; Figure 2(b)).

### 3.4. Gut Microbiota Community Composition

**Figure 3:**
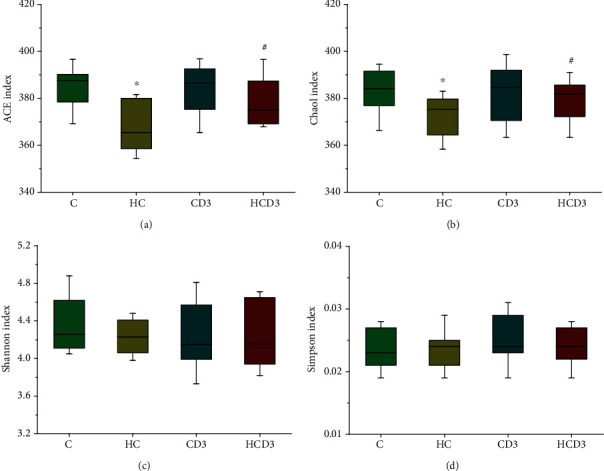
Analysis of the alpha diversity of gut microbiota among C57BL/6J mice on different diets. The richness was estimated by (a) the ACE index and (b) the Chao1 index, and the diversity was estimated by (c) the Shannon index and (d) the Simpson index. ^∗^*P* < 0.05 in comparison with group C, ^#^*P* < 0.05 in comparison with group HC. Data are represented as the mean ± SD (*n* = 5).

To elucidate whether vitamin D_3_ supplementation can change gut microbiota diversity and composition, the region of bacterial 16S rRNA gene V4 was evaluated in 20 cecal samples using Illumina MiSeq sequencing, producing 962,597 effective sequences. Figures 3(a)−3(d) show the calculation of the alpha diversity indices (ACE, Chao1, Shannon, and Simpson). The ACE and Chao1 indices of group HC were significantly lower than those of group C (*P* < 0.05), but they were much higher in the HCD3 group when compared with the HC group (Figures 3(a) and 3(b)). However, the Shannon and Simpson indices of the gut microbiota community in the four groups were not significantly different (Figures 3(c) and 3(d)).

**Figure 4:**
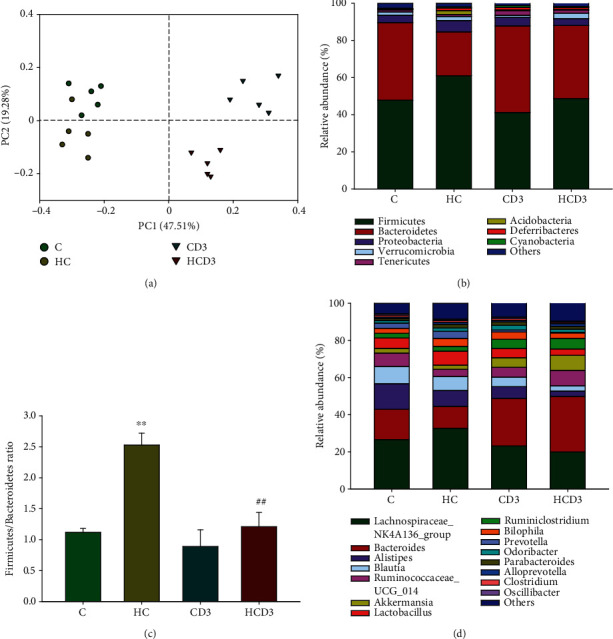
The impact of vitamin D_3_ supplementation on gut microbiota community composition. (a) Unweighted UniFrac-based PCoA plots illustrating differences in gut microbiota among groups (*β*-diversity); (b) relative abundance of gut microbiota (phylum level); (c) Firmicutes/Bacteroidetes ratio; (d) relative abundance of gut microbiota (genus level). The relative abundance of gut microbiota was calculated by averaging the data of the five replicates within each group. ^∗∗^*P* < 0.01 in comparison with group C, ^##^*P* < 0.01 in comparison with group HC.

Based on unweighted UniFrac distance matrices, the beta diversity was assessed by principal coordinates analysis (PCoA) (Figure 4(a)). Differences related to vitamin D_3_ supplementation were observed along the first principal coordinate (PC1), explaining 47.51% of the total variation; the C and HC groups clustered along PC2 separately, which accounted for another 19.28%. The PCoA results indicate that the gut microbiota composition was more sensitive to vitamin D_3_ supplementation than to a high-choline diet. Bacteroidetes, Proteobacteria, and Firmicutes were the main phyla in all groups at the phylum level, accounting for 90.65-93.57% of the gut microbiota community. A relatively higher abundance of Firmicutes was observed in the HC group (61.02%) than in either the C (47.82%) or HCD3 (48.65%) groups (*P* < 0.05), while the relative abundance of Bacteroidetes was much lower in the HC group (23.57%) than in either the C (41.71%) or HCD3 (39.34%) groups (*P* < 0.01; Figure 4(b)). The relative abundance of Proteobacteria among the groups was not significantly different. The Firmicutes/Bacteroidetes (F/B) ratio in the HC group (2.53 ± 0.19) was significantly larger than that in the C group (1.12 ± 0.06; *P* < 0.01), and the ratio was almost restored in the HCD3 group (1.21 ± 0.23; Figure 4(c)). At the genus level, *Bacteroides*, *Alistipes*, *Blautia*, and *Ruminococcaceae*_*UCG_014* were lower in the HC group in comparison with those in the C group. Vitamin D_3_ supplementation reversed the choline-induced changes in *Bacteroides* and *Ruminococcaceae*_*UCG_014*. Furthermore, significant increases in *Akkermansia* and *Ruminiclostridium* were found in the HCD3 group in comparison with those in both the C and HC groups (Figure 4(d)).

**Figure 5:**
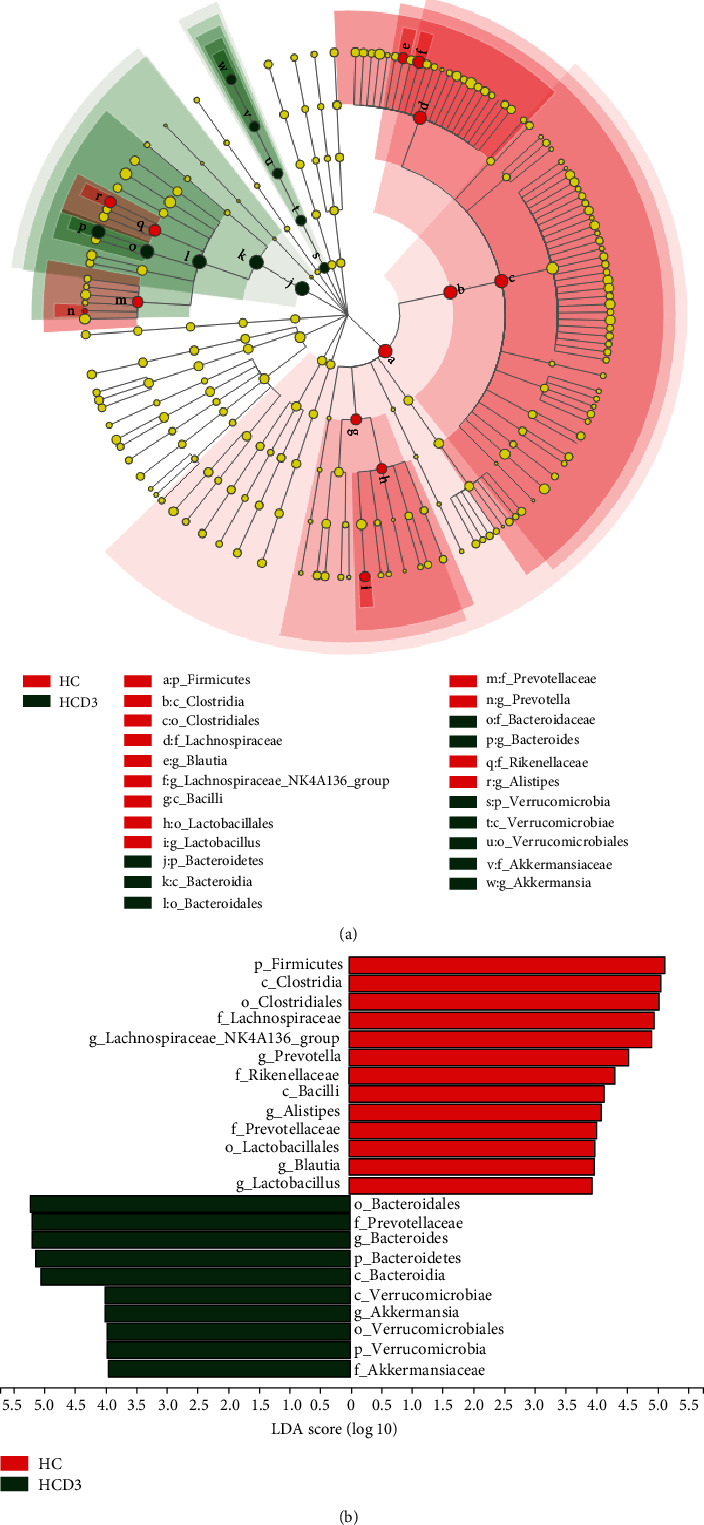
Taxonomic cladogram and LDA scores for LEfSe analysis of gut microbiota in the HC and HCD3 groups. (a) Taxonomic cladogram represents significant differences in taxa between groups (*n* = 5). Taxonomic levels from phylum to genus are represented by rings. (b) LDA scores for differentially abundant taxa between groups (taxa with an LDA significant threshold ≥ 4 are shown).

LEfSe analysis was performed to distinguish bacterial taxa between groups C and CD3 (Figures S1(a) and S1(b)) and between groups HC and HCD3 (Figures 5(a) and 5(b)) in order to evaluate whether there was any influence of vitamin D_3_ on gut microbiota. The LEfSe analysis results show that *Lachnospiraceae*_*NK4A136_group* (from phylum to genus), *Prevotella* (from family to genus), *Alistipes* (from family to genus), *Blautia* (from phylum to genus), and Lactobacillales (from phylum to order) were significantly more abundant in the HC group, while *Bacteroides* (from phylum to genus) and *Akkermansia* (from phylum to genus) were enriched in the HCD3 group.

### 3.5. Plasma Level of TMAO Is Associated with Gut Microbial Taxa

**Figure 6:**
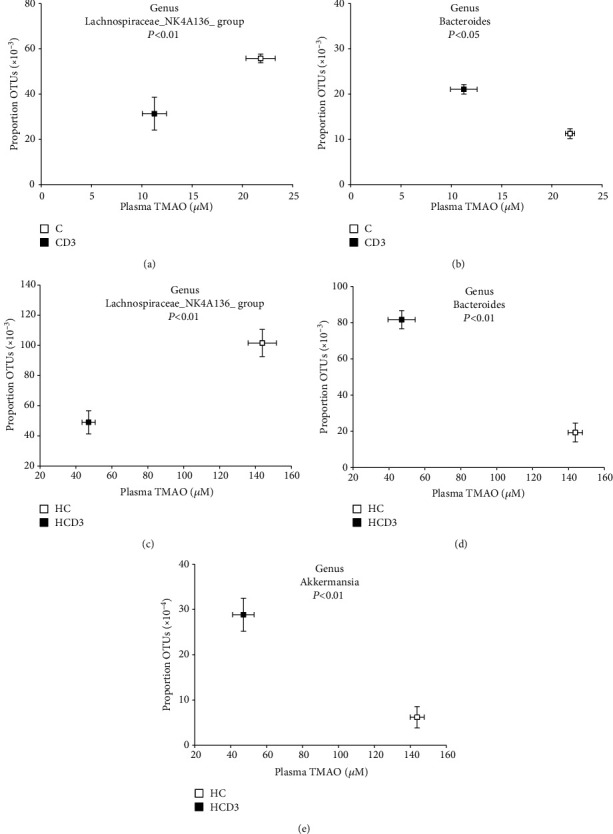
The gut microbiota composition at the genus level is associated with the plasma TMAO level. Data are represented as the mean ± SD (*n* = 5).

To further identify correlations between plasma TMAO level and gut microbiota, Spearman correlation analysis was conducted. The results show that plasma TMAO levels were positively correlated with *Lachnospiraceae*_*NK4A136_group* (*r* = 0.78, *P* < 0.01) and negatively correlated with *Bacteroides* (*r* = −0.53, *P* < 0.05) in groups C and CD3 (Figures 6(a) and 6(b)). The plasma TMAO level was positively correlated with *Lachnospiraceae*_*NK4A136_group* (*r* = 0.46, *P* < 0.01) and negatively correlated with *Bacteroides* and *Akkermansia* (*r* = −0.66, *P* < 0.01; *r* = −0.25, *P* < 0.01) in groups HC and HCD3 (Figures 6(c)−6(e)).

## 4. Discussion

Prior studies have indicated that the plasma TMAO level is positively correlated with an increased risk of CVD and all-cause death [17]. Clinical studies have revealed a link between TMAO and CVD prevalence among a multiethnic population [18]. In addition to CVD, TMAO has been linked to nonalcoholic fatty liver disease, diabetes, chronic kidney disease, and colorectal cancer [19, 20]. Thus, reducing the plasma TMAO level is considered a novel method to treat and prevent CVD and other diseases. Abundant evidence indicates that the production of TMAO is mediated by gut microbiota. Using broad-spectrum antibiotics to delete the gut microbiota could eliminate choline-induced TMAO production and the development of AS in both mice and humans [21, 22]. In the present study, plasma TMA and TMAO levels were greatly reduced in the antibiotic-treated group (HCD3A), which is consistent with previous observations. These results further support the obligatory role of gut microbiota in the formation of TMA and TMAO. However, antibiotics are not an ideal treatment since long-term application may have other adverse consequences such as organ damage and drug resistance.

Both clinical and animal studies have shown that FMO3 oxidizes TMA to TMAO in the liver, and its expression is the main regulator of the TMAO level [23]. FMO3-knockout mice have a reduced plasma TMAO level and do not develop AS but have aggravated liver endoplasmic reticulum stress and inflammation [24]. Notably, patients with loss-of-function mutations in FMO3 often suffer from trimethylaminuria (fish odor syndrome) [25]. Our results indicate that the difference in liver FMO3 expression among different diet groups was not statistically significant. However, the TMAO level in the HC group was much higher than that in the HCD3 group. Therefore, vitamin D-induced decrease in plasma TMA and TMAO levels was not due to the regulation of FMO3 in the liver, and we hypothesized that the reduction in plasma TMA and TMAO levels can be attributed to the effect of changes in the composition of gut microbiota on the conversion of choline to TMA. Bennett et al. demonstrated that FMO3 expression is genetically regulated and that farnesoid X receptor (FXR) is a crucial regulator of the FMO3 level [26]. Liver FMO3 expression is gender-dependent in mice, and testosterone represses the expression of FMO3 in a male mouse liver, whereas females exhibit a high FMO3 level [27]. However, the regulated liver FMO3 expression and activity exact underlying mechanisms need to be further elucidated.

The present study provides novel findings that vitamin D supplementation can reduce the levels of plasma TMA and TMAO through the regulation of gut microbiota composition and may serve as a novel method for the prevention of AS and the resulting CVD. The results of *α*-diversity analysis show that the ACE and Chao1 indices of group HC were much lower than those of group C, indicating that a high-choline diet decreased the richness of the gut microbiota community. However, vitamin D can improve the richness of gut microbiota caused by high-choline diet. In addition, the results of PCoA analysis demonstrate a significant separation between groups C and CD3 and between groups HC and HCD3, confirming that vitamin D supplementation can change the structure of gut microbiota. Our study found that Firmicutes was significantly more abundant and Bacteroidetes significantly less abundant in group HC in comparison with groups C and HCD3. This is consistent with the increased plasma TMA and TMAO levels and is in accordance with earlier reports showing that the ability to produce TMA is strong in Firmicutes but very weak in Bacteroidetes [28]. Moreover, the F/B ratio of the HC group was markedly larger than that of the C group but was significantly decreased after vitamin D supplementation (HCD3). A high F/B ratio has previously been linked to an increased risk of obesity and metabolic diseases associated with AS [29]. Cui et al. demonstrated that patients with coronary heart disease had fewer Bacteroidetes and Proteobacteria, and more Firmicutes and Fusobacteria, as compared with healthy controls [30]. A low F/B ratio has been associated with healthy gut microbiota and could possibly be helpful in preventing coronary atherosclerosis. The results of LEfSe analysis show that *Prevotella*, *Lachnospiraceae_NK4A136_group*, *Blautia*, and *Alistipes* were enriched in the HC group, while *Bacteroides* and *Akkermansia* were enriched in the HCD3 group. We also found that the plasma TMAO level was significantly negatively correlated with the abundance of *Akkermansia* and *Bacteroides*. Karlsson et al. sequenced the fecal metagenome of symptomatic patients with AS and control individuals and observed that *Bacteroides* was more abundant in the control group [31]. Animal studies have shown similar results [32]. *Akkermansia* has been demonstrated to exert antiatherosclerotic effects by reversing metabolic disorders, modulating inflammation, and maintaining the integrity of the gut barrier [33]. An *in vitro* study by Romano et al. showed that *Akkermansia muciniphila* did not have the ability to produce TMA from choline [34]. Our results show that vitamin D supplementation can reduce the plasma TMAO level, which may be partly attributed to the increase in *Bacteroides* and *Akkermansia*, and developing specific inhibitors targeting gut microbiota TMAO production will be more practical.

Vitamin D can regulate the gene expression of cathelicidin antimicrobial peptide (CAMP), thereby mediating the antibacterial action of CAMP. The effect of oral vitamin D on human gut microbiota is shown to reduce opportunistic pathogens and increase bacterial richness [35]. Vitamin D exerts its biological activity by binding to vitamin D receptor (VDR), and VDR is most expressed in CD8^+^ T cells, when compared with other immune cells [36]. In addition, there is increasing evidence that VDR is a key component in maintaining gut intestinal barrier function and preventing dysbiosis and has a role in reducing inflammation [37]. We speculated that reducing the inflammatory environment through vitamin D could decrease competitive advantages of opportunistic pathogens such as *Prevotella*, enabling beneficial bacteria such as *Bacteroides* and *Akkermansia* to defeat opportunistic pathogens, thus regulating the structure of gut microbiota and increasing the richness of gut microbiota. However, the function of VDR and its role in gut microbiota composition and function regulation have not been fully elucidated, and more researches are needed.

According to a previous study, 3,3-dimethyl-1-butanol (DMB) present in olive oil, grape seed oil, and red wine can inhibit TMA lyase, reducing TMA and TMAO levels [38]. In addition, DMB reduces the TMA- and TMAO-associated microbial taxa, choline-induced foam cell formation, and atherosclerotic plaque development. Moreover, recent studies have shown that direct regulation of gut microbiota through probiotics, phytoalexin, resveratrol, a Mediterranean diet, and other interventions reduces TMAO levels in mice and thus may be considered as novel avenues for the prevention and treatment of CVD [21, 39, 40]. Further researches regarding the function and mechanism are required to clarify the association between gut microbiota and CVD. Nevertheless, our study shows that vitamin D supplementation is a promising method to reduce the plasma TMAO level, with potential for the prevention and treatment of CVD, especially in individuals who have a high risk of CVD but do not currently require prescription drugs.

## 5. Conclusion

Vitamin D supplementation reduced the levels of choline-induced TMA and TMAO by regulating the composition of gut microbiota. A negative correlation was observed between the plasma level of TMAO and the abundance of *Bacteroides* and *Akkermansia*. Our work provides novel insight into the prevention and treatment of CVD with vitamin D.

## Figures and Tables

**Table 1:** Food intake, body weight, and serum TC, TG, LDL-C, HDL-C, and 25(OH)D levels.

Parameters	C (*n* = 9)	HC (*n* = 9)	CD3 (*n* = 10)	HCD3 (*n* = 8)	HCD3A (*n* = 9)
Food intake (g/day)	3.45 ± 0.29	3.59 ± 0.17	3.36 ± 0.15	3.31 ± 0.13	3.23 ± 0.21
Body weight (g)	23.27 ± 1.08	22.63 ± 0.81	23.42 ± 1.75	22.86 ± 1.93	21.35 ± 2.43
TC (mmol/L)	2.83 ± 0.45	3.27 ± 0.33	2.99 ± 0.37	3.08 ± 0.25	3.21 ± 0.19
TG (mmol/L)	1.39 ± 0.16	1.68 ± 0.07	1.33 ± 0.21	1.71 ± 0.28	1.57 ± 0.14
LDL-C (mmol/L)	1.62 ± 0.37	2.12 ± 0.11	1.74 ± 0.14	2.04 ± 0.12	1.82 ± 0.32
HDL-C (mmol/L)	2.39 ± 0.13	2.05 ± 0.24	2.26 ± 0.29	2.47 ± 0.16	1.94 ± 0.26
25(OH)D (ng/mL)	26.38 ± 3.41	27.71 ± 5.95	62.03 ± 9.26^∗∗^	59.45 ± 4.38^∗∗^	51.07 ± 8.02^∗∗^

Data are represented as the mean ± SD. Significant differences between C and CD3, HC and HCD3, and HC and HCD3A were determined by one-way ANOVA. ^∗∗^*P* < 0.01.

## Data Availability

All data are included within the article and supplementary documents.

## Supplementary Materials

Supplementary Materials Figure S1: Taxonomic cladogram and LDA scores for LEfSe analysis of gut microbiota in groups C and CD3.
