# Supplementary material for: Vitamin D Decreases Plasma Trimethylamine-N-oxide Level in Mice by Regulating Gut Microbiota
Source: Biomed Res Int. 2020 Oct 5;2020:9896743. doi: 10.1155/2020/9896743 (PMC7558778; doi:10.1155/2020/9896743)
Supplement: Supplementary Materials — Figure S1: Taxonomic cladogram and LDA scores for LEfSe analysis of gut microbiota in groups C and CD3. [file 9896743.f1.pdf]

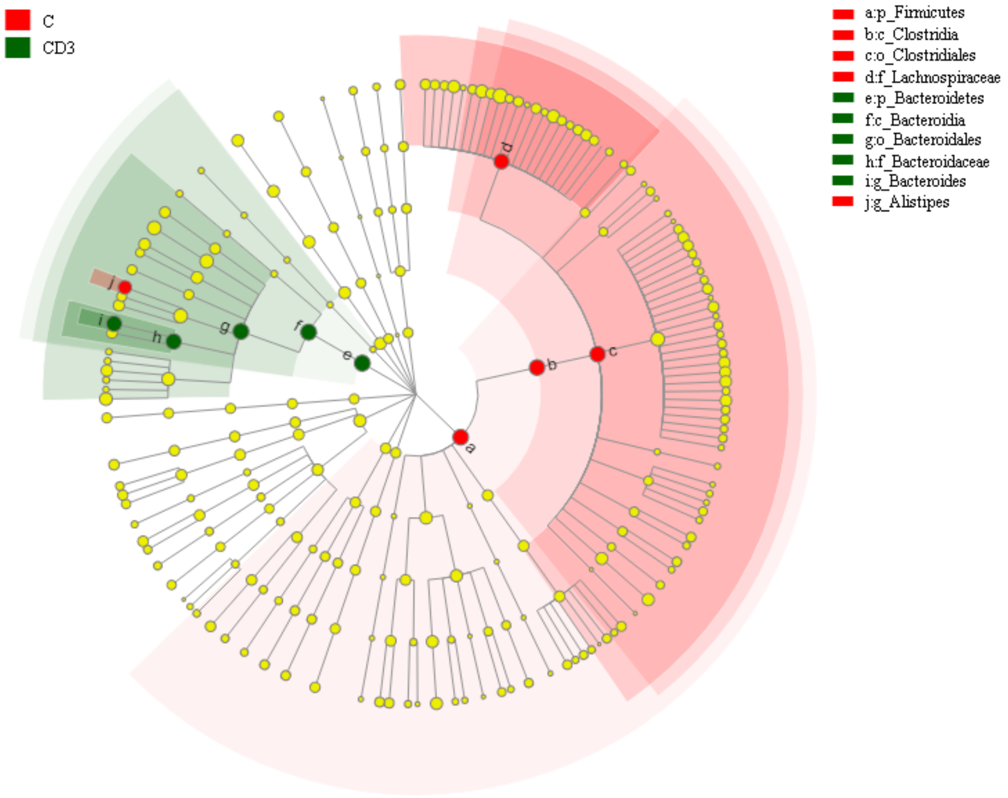

(a)

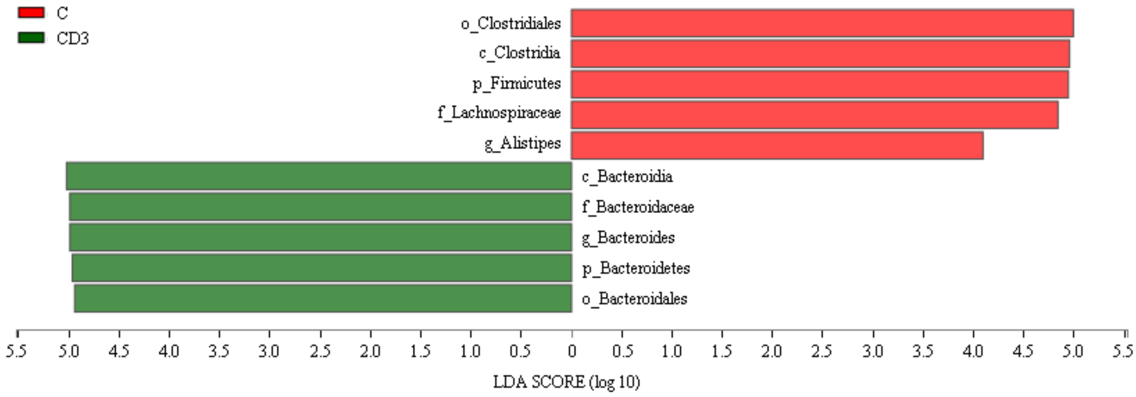

(b)

FIGURE S1: Taxonomic cladogram and LDA scores for LEfSe analysis of gut microbiota in the C and CD3 groups. (a) Taxonomic cladogram represents significant differences in taxa between groups (n=5). Taxonomic levels from phylum to genus are represented by rings. (b) LDA scores for differentially abundant taxa between groups (taxa with an LDA significant threshold≥4 are shown).
